# Correction: Mohammed et al. Assessment of Knowledge, Attitude, and Practice towards Tuberculosis among Taif University Students. *Healthcare* 2023, *11*, 2807

**DOI:** 10.3390/healthcare12020180

**Published:** 2024-01-11

**Authors:** Eilaf A. Mohammed, Huriyyah A. Alotaibi, Joud F. Alnemari, Meznah S. Althobiti, Shumukh S. Alotaibi, Ashraf A. Ewis, Azza A. K. El-Sheikh, Sayed F. Abdelwahab

**Affiliations:** 1 College of Pharmacy, Taif University, Taif 21944, Saudi Arabia; s43800156@students.tu.edu.sa (E.A.M.); s43804056@students.tu.edu.sa (H.A.A.); s43800798@students.tu.edu.sa (J.F.A.); s43810784@students.tu.edu.sa (M.S.A.); s43802037@students.tu.edu.sa (S.S.A.); 2 Department of Public Health, Faculty of Health Sciences, Umm Al-Qura University, Makkah 21912, Saudi Arabia; aaewis@uqu.edu.sa; 3 Basic Health Sciences Department, College of Medicine, Princess Nourah Bint Abdulrahman University, P.O. Box 84428, Riyadh 11671, Saudi Arabia; aaelsheikh@pnu.edu.sa; 4 Department of Pharmaceutics and Industrial Pharmacy, College of Pharmacy, Taif University, Taif 21944, Saudi Arabia

In the original publication [1], a mistake was published in Table 6. The numbers and percentages for the marital status “single” were incorrect. The correct numbers are shown below in the updated version of Table 6. The authors state that the scientific conclusions are unaffected. This correction was approved by the Academic Editor. The original publication has also been updated.

## Figures and Tables

**Table 6:** Assessment of the knowledge score of the study participants towards TB.

Variable	Knowledge Score N (%)			
	Excellent *n* = 165 (19.4%)	Good *n* = 386 (45.5%)	Poor *n* = 298 (35.1%)	*p*-Value
**Gender**				0.142
Male	53 (19.7%)	110 (40.9%)	106 (39.4%)	
Female	112 (19.3%)	276 (47.6%)	192 (33.1%)	
**Age (years)**				**<0.001**
18–20	32 (10.3%)	153 (49.2%)	126 (40.5%)	
21–24	125 (25.6%)	208 (42.5%)	156 (31.9%)	
≥25	8 (16.3)	25 (51.0%)	16 (32.7%)	
**Nationality**				0.292
Saudi	161 (19.5%)	378 (45.8%)	286 (34.7%)	
Non-Saudi	4 (16.7%)	8 (33.3%)	12 (50.0%)	
**Type of College**				**<0.001**
Medical Colleges ^#^	134 (31.3%)	197 (45.9%)	98 (22.8%)	
Science and Engineering Colleges ^#^	17 (8.0%)	97 (45.5%)	99 (46.5%)	
Humanities, Education, etc. ^#^	14 (6.8%)	92 (44.4%)	101 (48.8%)	
**Educational level**				**<0.001**
1st and 2nd	29 (9.8%)	141 (47.8%)	125 (42.4%)	
3rd and 4th	72 (19.5%)	168 (45.7%)	128 (34.8%)	
5th and 6th and above	64 (34.4%)	77 (41.4%)	45 (34.2%)	
**Marital status**				0.916
Single	157 (19.5%)	366 (45.5%)	281 (35.0%)	
Married +	8 (17.8%)	20 (44.4%)	17 (37.8%)	
**Smoking**				0.245
Non-smoker	151 (20.2%)	339 (45.4%)	257 (34.4%)	
Smoker *	14 (13.7%)	47 (46.1%)	41 (40.2%)	
**Do you know people who have/had TB?**				**0.02**
Yes	9 (15.3%)	37 (62.7%)	13 (22.0%)	
No	156 (19.7%)	349 (44.2%)	285 (36.1%)	

# Medical Colleges include Medicine, Dentistry, Pharmacy, and Applied Medical Sciences. Science and Engineering Colleges include Science, Engineering, Computer Science and Information Technology, Design and Applied Arts. Humanities, Education, Sharia and Administrative Colleges include Sharia and Regulations, Business Administration, Literature, Education, and Applied Colleges. Bold numbers indicate statistical significance. + is married plus Divorced/Widow, * is smokers and ex-smokers.
